# The State of Simulation in Emergency Medicine Residency Programs in the United States

**DOI:** 10.5811/westjem.42048

**Published:** 2025-10-21

**Authors:** Briana D. Miller, Charles Khoury, Jaron Raper, Lauren A. Walter, Andrew Bloom

**Affiliations:** * Oregon Health & Science University, Department of Emergency Medicine, Portland, Oregon; † University of Alabama at Birmingham, Department of Emergency Medicine, Birmingham, Alabama

## Abstract

**Introduction:**

Using simulation-based medical education has proven to be an effective instructional strategy both procedurally and clinically. Emergency medicine (EM) residency programs use simulation in a variety of ways and settings. Given the ongoing development of the field and the recent expansion of EM training programs, our objective was to assess the current state of simulation use in Accreditation Council for Graduate Medical Education (ACGME)-approved EM residency programs in the United States.

**Methods:**

We performed this cross-sectional national survey from July–September 2022. The survey was sent to the residency program directors of all 277 ACGME-accredited EM residency programs in the US. The survey focused on simulation use, technology, types of simulation (procedural vs case-based), barriers to growth, and overall sentiments regarding simulation in EM.

**Results:**

We attempted to contact 277 programs, successfully reaching 244. We received a total of 100 responses (36%). Nearly all responding programs reported access to a dedicated sim center (95.8%), with available high-fidelity manikin simulators (93%) and task trainers (90%). Most programs engage in simulation didactics monthly (50%), followed by more than monthly (22%) and quarterly (19%). Barriers to simulation implementation included funding, simulation lab availability, and equipment. Programs frequently used simulation to perform the majority of rare but required procedures, and about half of the programs responding reported simulation fellowship-trained faculty on staff.

**Conclusion:**

Simulation education is an important aspect of EM residency and training. Most residency programs reported dedication and resources to developing and integrating simulation into their curriculum. There is likely room for its further use in residency program training in the coming years as residency programs continue to expand.

## INTRODUCTION

Simulation-based medical education is an effective educational tool in emergency medicine (EM) training that incorporates experiential learning and deliberate practice to create an engaging learning environment. Simulation encompasses a broad spectrum of activities, which can include high-fidelity clinical simulations, procedural training using task trainers, difficult conversations with standardized patients, in-situ simulations in the actual clinical environment, and, more recently, virtual reality scenarios. Technology-enhanced simulation training has been associated with improved learning outcomes compared to other educational methods.^1^ Simulation-based education plays a particularly important role in EM residency training, where physicians must become proficient in managing high-acuity, low-frequency clinical situations and procedures that may not occur naturally during three or four years of residency.^2^ However, there is significant variability in the use of simulation in Accreditation Council for Graduate Medical Education (ACGME)-accredited EM residency training programs nationally.

The most recent national survey regarding the use of simulation in United States EM residency programs was by Okuda et al in 2008 who reported that 91% of programs used simulation, with 43% of programs using simulation more than 10 hours per year.^3^ This showed a significant increase from the prior survey performed in 2003 that reported only 29% of EM programs were using high-fidelity simulation.^4^ Since then, there has not been a re-evaluation of simulation use in US EM residency programs, although a 2015 survey of pediatric EM fellowships reported using simulation in 97% of programs.^5^ In 2011, Heitz et al reported that 79% of EM clerkships were incorporating simulation into their curriculum for third- and fourth-year medical students.^6^ A more recent survey of Canadian EM residency programs found 100% of programs reported using simulation for resident education, with a median of 20 dedicated hours per year.^7^,^8^ It is unclear whether these data can be directly translated to US training programs, as Canada’s EM programs have been encouraged to transition to a competency-based education residency model that may inherently use simulation more. 9 Simulation fellowship programs have also exhibited significant growth, which may be an indirect marker of an increase in demand for simulation-trained faculty.^10^–^12^

Over the past 15 years, the landscape of EM resident training has evolved dramatically, especially in an era that confronted a global pandemic and record emergency department (ED) boarding. Anecdotally, the use of simulation education has increased in EM residency training programs over this time, but data are limited on the current amount and methods of implementation. Understanding the role of simulation in EM residencies across the country may allow programs to standardize simulation curricula, motivate formal simulation facilitation training, and offer opportunities for expansion of the learning modality in the context of EM. Our objective in this study was to provide an update of the use of simulation in EM resident training programs in the US.

## METHODS

### Study Design and Population

Approval for this project was obtained from the institutional review board (IRB) (IRB-300008219). We conducted a national survey of EM residency program directors (PD). The survey tool was an expansion and update of earlier surveys administered to EM programs.^3^–^6^

### Survey Content and Administration

Surveys were sent to 277 PDs of EM residency programs that participated in the 2022 match, as identified by the Council of Residency Directors in EM directory and the National Resident Matching Program.^13^ Surveys were distributed via email in August 2022. Each program was solicited to complete a single survey. The email provided a brief introduction regarding the purpose of the survey, the title and role of the survey solicitors, and documentation of IRB approval. A direct link was provided in the email to a web-based survey tool (Qualtrics International Inc, Provo, UT). A second email was sent to the same distribution list in October 2022, as a final solicitation and reminder.

**Population Health Research Capsule**

What do we already know about this issue?

*Anecdotally, simulation use in emergency medicine training programs has increased over the past decade. However, few data exist to support or quantify this increase.*

What was the research question?

*We sought to assess the current state of simulation use in ACGME-approved EM residency programs in the United States.*

What was the major finding of the study?

*75% of programs use simulation at least monthly, reflecting a notable increase compared to 43% using simulation greater than 10 hours per year in a 2008 survey data.*

How does this improve population health?

*Simulation-based education improves patient safety, and its growing use in EM residencies reflects a strong commitment to safer, high-quality clinical training.*

The survey was developed via an iterative process. After initial development, we piloted the survey at our institution with an EM PD and two assistant PDs. Using feedback and suggestions for edits, the revised survey was piloted once more with one PD at a different institution. The final version was distributed to our list of PDs as shown in Addendum 1. The final updated instrument contained program demographic, yes/no, and multiple-choice questions related to the availability and use of simulation in EM residency training. Additional questions pertained to the availability of simulation fellowship-trained faculty, simulation scholarly tracks and research activities, and the accreditation of available simulation centers .

As in prior assessments, we defined a manikin-based, high-fidelity simulator as a computerized full-body robot-manikin. A partial task simulator was defined as specialized equipment for procedures or skills that go beyond typical static trainers. Examples include simulators for central lines, venous access, chest tube placement, bronchoscopy, ultrasound, birthing, and trauma. We defined screen-based computer simulation as case simulations conducted through an interactive computer interface. A tabletop-based simulation was defined as a low-fidelity, discussion-based scenario designed to apply knowledge and/or assess policies, plans, or procedures.^14^

Efforts to minimize non-response bias included sending reminder requests; however, funding constraints prevented us from offering financial incentives to PDs for completing the survey. Additionally, we were unable to calculate non-response bias due to not tracking survey response dates.

### Data Analysis

We analyzed results using simple frequency tabulations; chi-square analysis was employed to compare current and previous survey data.

## RESULTS

Of the 277 programs surveyed, we successfully contacted 244, and 100 PDs completed the questionnaire. This corresponds with a response rate of 36% based on all programs surveyed and 41% based on programs successfully contacted. For consistency, we use the 36% response rate throughout the paper, in alignment with the American Association for Public Opinion Research definition of minimum response rate.^15^ Programs from all geographic regions responded (Table 1). Most of the programs that responded were comprised of more than 28 total residents (69.5%).

With regard to equipment, personnel, and resources, approximately half (49.5%) of respondents reported simulation fellowship-trained faculty on staff, most frequently comprised of one (20%) or two (17.9%) individuals. Most (95.8%) programs reported access to a simulation center, 34.7% of which were reportedly accredited by the Society for Simulation in Healthcare. Larger residency programs (those with more than 27 residents) were more likely to have more simulation equipment (*P* > .05). Simulation was most often used monthly (52.6%) or more than once per month (23.2%). A smaller number of programs reported using simulation 3–11 times per year (20%), while one program (1.1%) reported using it 2–3 times per year. Most respondents (74.7%) also reported use of simulation curriculum or education for medical students on their EM rotation.

Simulation was used most frequently to address resuscitation skills, airway management, disease-specific management (eg, myocardial infarction, arrhythmia), procedural skills, professionalism, teamwork, and error avoidance. In most responding programs (85.2%), simulation was used to fulfill training requirements for rare ACGME-mandated procedures, such as pericardiocentesis, cardiac pacing, and cricothyrotomy, **in at least half of the required cases**. In contrast, more routine required procedures, such as central venous line placement, intubation, and lumbar puncture, were simulated **in fewer than 20% of cases** at most (77.9%) programs. Over half of programs (60%) reported engagement in simulation-based research resulting in poster or abstract presentation (55.8%) and/or peer-reviewed journal publication (40%). Ongoing reported barriers to incorporation of simulation included lack of funding, lack of lab or equipment access, and lack of faculty expertise or availability.

## DISCUSSION

Research on the use of simulation-based medical education in US EM residency programs has been sparse since the 2008 study by Okuda et al. Our findings suggest a notable increase in simulation use, with 75% of programs now reporting simulation sessions at least once per month compared to just 42% of programs in 2008, which reported >10 hours of simulation annually.^3^ Nearly all programs reported using simulation in general, while more than 90% reported using both manikin-based (97.9%) and task trainer-based (94.7%) simulation for procedural teaching. The 2008 data from Okuda documented that only 60% of respondents reported using task-trainer simulators, while 85% used manikin simulators.^3^

Over half of programs (58.9%) reported access to screen-based simulators compared to 14% in the 2008 survey.^3^ The reason for this increase is likely multifactorial, including the increased accessibility and decreased cost of this technology as it has evolved, and potentially due to the need for novel educational methods during the COVID-19 pandemic where traditional teaching modalities were not safe. A future direction of research could involve re-evaluating the prevalence of screen-based simulation activities now that most institutions have returned to in-person educational activities.

We surmise that the increase in simulation use is largely due to the growing recognition of its benefits in medical education, supported by evidence from multiple studies. Most importantly, extensive research has demonstrated improvements in patient safety and the quality of care as a result of simulation-based education.^16^–^18^ The high frequency of using simulation to teach rare clinical procedures (and fulfill ACGME procedure requirements) is not surprising, as most residents likely do not experience enough of those rare procedures in clinical practice alone to be competent and confident. Most respondents indicated access to a simulation center, which is encouraging, although that response rate may have been affected by the large number of responses obtained from university-based EM residency programs.

Simulation appears to be a priority for medical student rotations in EM as about three-quarters of respondents indicated that their students participate regularly in simulation. We surmise that simulation can help combat students’ inconsistent opportunities to experience diverse clinical scenarios, given an often relatively short EM rotation. During the COVID-19 pandemic when students had less exposure to patients, simulation was recognized as a valuable tool to develop clinical skills previously taught exclusively at bedside. 19 Although students have now reintegrated into in-person clinical practice, the value of simulation as an educational tool appears to be persistent.

About half (49.5%) of responding programs reported having fellowship-trained simulation faculty on staff. While this may represent an increase in simulation-trained faculty and is encouraging for the field of medical simulation, it also highlights a need for training materials and support for non-fellowship-trained faculty at the other half of institutions that are also using simulation-based education. Lack of faculty expertise was also cited as a major barrier to simulation in responding programs, further underscoring the need for continued facilitation training. Anecdotally, there are reports of some facilitators who have not had formal simulation training but, through years of experience, have honed skills that have led them to become exceptional educators. However, the International Nursing Association for Clinical Simulation and Learning (INACSL) Healthcare Simulation Standards of Best Practice suggest that all simulation facilitators should possess “specific skills and knowledge in simulation pedagogy.”^20^ Additionally, only about a quarter (27.4%) of programs reported having a simulation fellowship or simulation scholarly track available to residents. This represents an opportunity for programs to expose their residents to simulation theory and facilitation earlier in training to spur their interest and expertise in medical education, debriefing skills, and different applications of simulation.

Major barriers to incorporating simulation included lack of funding, limited access to simulation materials or facilities, and lack of faculty availability and expertise. Not surprisingly, these findings closely mirror those cited by Okuda in 2008. 3 There is a clear need for increased access to simulation training and facilitation resources as well as support for increased fellowship opportunities. As simulation plays an increasing role in most programs’ curricula, increased departmental and institutional support will be necessary. Alternate revenue streams such as grants may also be explored to combat some of these barriers. There is also a growing body of free open-access simulation cases online.

## LIMITATIONS

The most prominent limitation is the survey response rate of 36%, not an uncommon interpretive barrier in survey-based projects. Similar survey-based studies targeting medical education program directors report response rates in the 49.5–54% range.^21^,^22^ While our response rate of 36% may introduce significant response bias, the diversity of respondents may still provide valuable insights into current practices.

We suspect multiple factors contributed to the low response rate of our survey. Program directors receive a multitude of emails daily so they could have missed the survey, or their contact information may have changed since the list was generated. Thirty-three of the emails sent were “undeliverable” or “invalid,” which limited responses from those programs where alternative contact information could not be located. Five of the surveys were only partially completed and, thus, excluded. Any survey is subject to response bias, and of the responding programs, there was a trend toward larger, academic programs. This likely biases the results as these programs are more likely to have funding and resources for simulation.

Due to the low response rate, our sample is likely subject to an increased risk of selection bias. Specifically, programs with robust simulation programs may have been more motivated to complete the survey, whereas programs without a strong simulation curriculum were less likely to participate. This response pattern could result in an over-representation of programs with established simulation initiatives, artificially inflating the reported prevalence and perceived impact of simulation programs among respondents.

In an effort to include all ACGME-accredited EM programs, the survey was sent to PDs, not specifically simulation faculty, which could have affected some nuances of responses. To our knowledge, there is not a current centralized, comprehensive list of simulation-trained or interested faculty at ACGME-accredited residency programs. There may be an opportunity for development of such a database through national organizations such as the Society of Academic Emergency Medicine Simulation Academy or the Society for Simulation in Healthcare Emergency Medicine Interest Group.

Another limitation of our results is that we collected data on simulation frequency in terms of number of sessions per year rather than in hours spent in simulation. For example, a program offering a four-hour simulation session once per month might be using simulation much differently than a program offering a one-hour simulation once per month, but both programs may have selected “monthly” to characterize their usage. This makes our results difficult to compare directly to prior studies that reported simulation use in hours. Future surveys should consider including this specific variable to better compare simulation curricula longitudinally.

## FUTURE DIRECTIONS

While this survey highlights the increase in use of simulation-based education in EM residency programs, it also highlights the variability in the methods and facilitation of simulation activities. It has recently been suggested that simulation be formalized as a core ACGME requirement; this survey may mobilize increased engagement in this initiative. 23 Further comparisons between programmatic and regional differences and educational outcomes would be useful to prioritize funding and equipment distribution. Formalizing the role of simulation in EM residency curricula may also encourage wider adoption of the INACSL Standards of Best Practice for Simulation.^24^ Future directions of research may also explore whether educational outcomes in simulation differ based on facilitator formal training or completion of a fellowship in simulation. Similarly, opportunities for faculty development and cost-effective and timely facilitator training could be explored.

While our findings show increased use of simulation in EM residency programs, and existing literature suggests a link to improved patient outcomes, further research is needed to determine whether increased simulation alone drives better educational results—or whether specific elements of a simulation curriculum are more effective. Such insights could help programs make more informed decisions about how to allocate time and resources.

Notably, this survey was conducted prior to the American Board of Emergency Medicine (ABEM) announcement of the upcoming change in certification examinations to clinical care cases and Objective Structured Clinical Examination cases. Simulation use in EM residency programs has the potential to change or expand in the next several years in conjunction with this change in ABEM examination policies. The increase in simulation use described in the survey results and the anticipated continued trajectory of the field also raise the question whether simulation should be an ACGME requirement for EM residency training programs.

## Supplementary Information



## Figures and Tables

**Table 1:** Characteristics of emergency medicine residency programs that participated in a survey of simulation-based medical education.

Residency Program Characteristic	Programs, n (%)
Region	
Midwest (IL, IN, IA, KS, MI, MN, MO, NE, ND, SD, OH, WI)	22 (23.2)
Southeast (AL, AR, FL, GA, LA, MS, NC, SC, TN, PR)	20 (21.1)
Southwest (AZ, CA, CO, HI, NV, NM, OK, TX, UT)	20 (21.1)
Mid-Atlantic (DE, DC, MD, NJ, PA, VA, WV)	16 (16.8)
Northeast (CT, ME, MA, NH, NY, RI, VT)	16 (16.8)
Northwest (AK, ID, MT, OR, WA, WY)	1 (1.1)
Residency size	
< 18	2 (2.1)
18–21	13 (13.7)
22–27	14 (14.7)
28–33	15 (15.8)
> 33	51 (53.7)
Primary residency type/affiliation	
University-based	54 (56.8)
Non-university based	24 (25.3)
County/public hospital	15 (15.8)
Military/other	2 (2.1)
Use of ANY simulation equipment	
Manikin simulators	93 (97.9)
Partial task simulators	90 (94.7)
Screen-based simulators	56 (58.9)
Tabletop simulators	59 (62.1)
Frequency of simulation didactics or education*	
1 time per year	0 (0.0)
2–3 times per year	1 (1.1)
4–11 times per year	19 (20.0)
Monthly	50 (52.6)
More than once a month	22 (23.2)

* Three missing responses.
