# Supplementary material for: The State of Simulation in Emergency Medicine Residency Programs in the United States
Source: West J Emerg Med. 2025 Oct 21;26(6):1530–5. doi: 10.5811/westjem.42048 (PMC12698156; doi:10.5811/westjem.42048)
Supplement: Supplementary file 1 [file wjem-26-1530-s001.docx]

**Addendum 1 – Survey sent to program directors**

**DEMOGRAPHICS:**

(1) What geographic location is your residency in?

[ ] Northeast (CT, ME, MA, NH, NY, RI, VT

[ ] Mid-Atlantic (DE, DC, MD, NJ, PA, VA, WV

[ ] Midwest (IL, IN, IA, KS, KY, MI, MN, MO, NE, ND, SD, OH, WI)

[ ] Southeast (AL, AR, FL, GA, LA, MS, NC, SC, TN, PR)

[ ] Southwest (AZ, CA, CO, HI, NV, NM, OK, TX, UT

[ ] Northwest (AK, ID, MT, OR, WA, WY)

(2) How many years is your residency program

[ ] 3 Years

[ ] 4 Years

(3) How many residents are in your program (as of 2022-2023 academic year)

­­­­­­­­

#_______­

(4) What category is your primary residency site?

[ ] University-based/academic

[ ] County/Public Hospital

[ ] Non-university-based

[ ] Military

[ ] Other

(5) Does your department have any Simulation Fellowship trained faculty on staff?

[ ] Yes

[ ] No

If Yes, how many ­­­_____

(6) Fellowship trained or not, does your department have faculty that dedicate their time to Simulation based education, training, and/or scholarly activities?

[ ] Yes

[ ] No

If Yes, how many ­­­_____

(7) Does your department have a Simulation Fellowship (as of 2022-2023 academic year)?

[ ] Yes

[ ] No

(8) Does your residency have a Simulation Scholarly Track (as of 2022-2023 academic year)?

[ ] Yes

[ ] No

(8a) if **no** to **#7 and #8** what are the reasons?

[ ] Lack of faculty experience/availability

[ ] Lack of resident interest

[ ] Chair or Program director preference

[ ] Funding

[ ] Residency does not have scholarly tracks

[ ] Lacks value compared to perceived effort

[ ] Do not feel it is beneficial or worthwhile

[ ] Other (specify)

(9) Does your department plan to develop advanced Simulation training/education by either a scholarly track or fellowship in the next 1-2 academic years?

[ ] Yes

[ ] No

[ ] Not sure

(10) Does your program have access to a Simulation Center?

[ ] Yes

[ ] No

(10a) If Yes, is it accredited by the SSH (Society for Simulation in Healthcare?)

[ ] Yes

[ ] No

[ ] Not sure

(11) Has your department engaged in Simulation research?

[ ] Yes

[ ] No

(11a) If yes, what has been done?

[ ] Presentation or poster at local/national/international conference

[ ] Peer-reviewed journal publication

**RESIDENCY CHARACTERISTICS**

(12) Does your residency engage in regular formal Simulation didactics or education?

[ ] Yes

[ ] No

(12a) If Yes, how often does this occur?

[ ] >1x/month

[ ] 1x/month

[ ] >3x/year

[ ] >1x/year

[ ] Yearly

(12b) Is this Simulation multidisciplinary (involving nurses, pharmacy, other specalities)

[ ] Yes

[ ] No

[ ] Sometimes, not always

(13) Does your residency have Simulation curriculum or education for medical students?

[ ] Yes

[ ] No

(14) Does your residency have access to a procedure lab/cadaver lab/procedural simulation equipment?

[ ] Yes

[ ] No

(14a) If yes does your program have difficulty obtaining access to a procedure lab or equipment?

[ ] Yes

[ ] No

(14b) If yes, why do you think there is difficulty?

[ ] Lack of faculty experience/availability

[ ] Chair or Program director preference

[ ] Funding

[ ] Time/equipment

[ ] Simulation lab availability

[ ] Interprofessional relationships

[ ] Other (specify)

(15) Does your residency engage in a formal procedural lab?

[ ] Yes

[ ] No

(15a) If Yes, how often does this occur?

[ ] >1x/month

[ ] 1x/month

[ ] >3x/year

[ ] >1x/year

[ ] Yearly

(16) ACGME requires no more than 30% of procedures (except for rare procedures – Pericardiocentesis, Cardiac pacing, Cricothyroidotomy) be performed within a simulated setting. On average what percentage of these (more common) procedures do you think are performed in a simulated setting in your program?

[ ] 30%

[ ] 20%-29%

[ ] 10%-19%

[ ] Less than 10%

(17) What percentage of rare procedures (Pericardiocentesis, cardiac pacing, Cricothyroidotomy) are performed in a simulated setting

[ ] 100%

[ ] ~75%

[ ] ~50%

[ ] ~25%

[ ] Less than 25%
